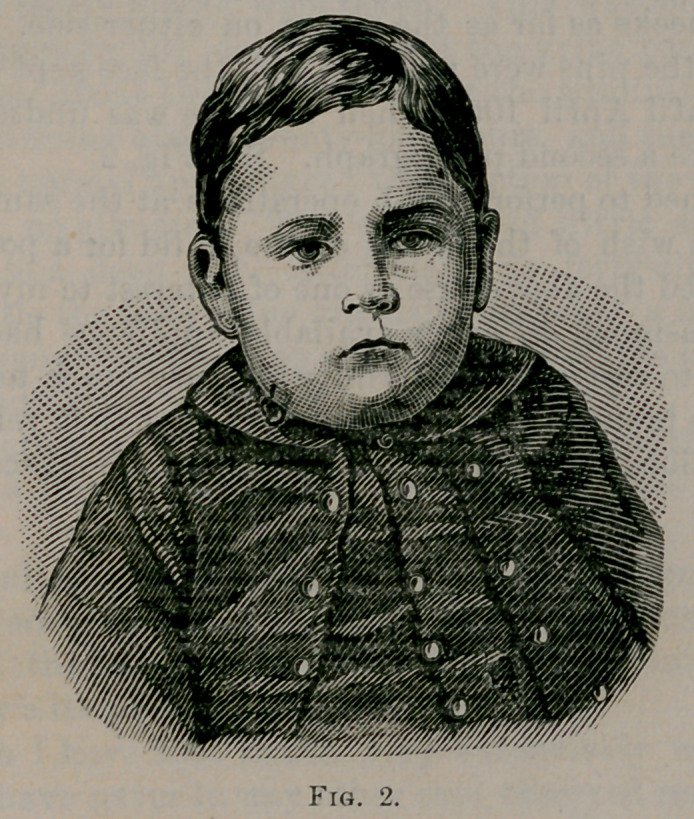# A Case of Congenital Torticollis, with Fissure of Upper Lip

**Published:** 1884-12

**Authors:** Jno. D. S. Davis

**Affiliations:** Birmingham, Ala.


### A CASE OF CONGENITAL TARTICOLLIS, WITH FISSURE OF UPPER LIP.

BY JNO. D. S. DAVIS, M.D., BIRMINGHAM, ALA.

On March 20, 1884, Willie A—, aged two years, of Talladega, Ala., was brought to my office for treatment of deformities which had existed from infancy. He was of healthy parents, and himself in good health other than trouble alluded to. I found, upon examination, the right sterno-cleido-mostoid muscle involved. Bringing about

FIG. 1.

this peculiar position of the head, as shown in Fig. 1, which was due to rotation of the head upon its axis, to allow the approximation of the origin, and insertion of this sterno-cleido-mostoid muscle and single hare lip of left side of upper lip extending into the nostril, I placed the head as nearly as possible in its normal position, carefully retaining it in that position. While the parts were thus kept on the stretch, I made additional pressure with my finger upon the lower fourth of the sterno-cleido-mostoid muscle (the part most tense), which immediately produced reflex contraction, pain and spasm. I had Mr. Scholze to take a photograph of the child, fig. 1; and at once proceeded to operate. The head of the child was placed as nearly as possible in a natural position, and retained by competent assistants, without the use of an anæsthetic. While the muscle was in this way made tense, or put upon the stretch, I introduced a curved tenotomy knife flatwise, through the integuments, on the inner side of muscle, about three-fourths of an inch above the sterno-clavicular articulation; carrying the knife through the tissues slowly, closely hugging the sternal head of the muscle, and then carrying the blade flatwise as introduced beneath the muscle to near the external or posterior side of the clavicular head of the

FIG. 2.

muscle; when I turned the cutting edge toward the muscle, and with a sawing motion severed both heads by one long incision through a single small puncture of the skin. The knife was then turned flatwise, and withdrawn as introduced, at the same time following the point of the blade of my instrument with the finger of my left hand, so as to press out all the blood, and to prevent air from entering the wound. The wound was then covered with a single strip of adhesive plaster, and the head held in position by passing a rubber strap under the axilla, and fastening the two ends of this strap to a linen roller bandage, parted around the head, and kept in position by a cross bandage over the head, connecting roller on either side. And right here I will say, that in retaining my head support I always fix my bandage around head in the way just described, in preference to using adhesive straps attached to the bandage and skin of forehead—thus allowing a removal at liberty, and avoiding the unpleasantness of the plaster. The support was used until April 1st, 1884, when it was removed, and the head remained in a perfectly normal position.

On April 1st, my friend Dr. Merrill anæsthetized the patient for me, and I paved both sides of the fissure in lip, and brought them together with two hare-lip pins, in the usual way. Then I drew the sides of the face as far forward as possible, and retained in that position by means of adhesive strips passed over the pins and attached to cheeks as far as the ramus on either side. At the end of fifty hours the pins were removed, and the face kept dressed with the plaster until April 10th, when his face was undressed for Mr. Scholze to take a second photograph. See Fig. 2.

I was inclined to perform both operations at the same time; but gave way to a wish of the father of the child for a postponement. I have reported the above case as one of interest to myself and my associates, inasmuch as every available appliance had been used on this child to correct the deformity but very much to no avail, ere the child was brought to my observation, and that relief was had by one operation, almost free from pain, and without scarcely a drop of blood.

## Figures and Tables

**Fig. 1. f1:**
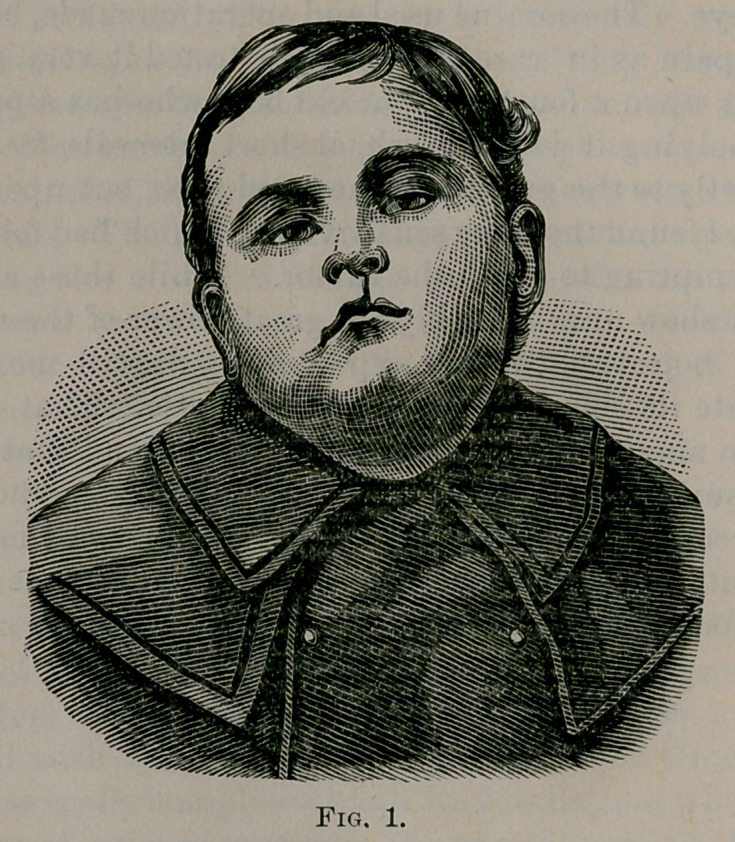


**Fig. 2. f2:**